# Chest Injury Evaluation and Management in Two Major Trauma Centers of Isfahan Province, IR Iran

**DOI:** 10.5812/atr.6542

**Published:** 2012-08-21

**Authors:** Mahdi Mohammadzadeh, Mehrdad Hosseinpour, Azadeh Sadat Mirzadeh, Hoda Jazayeri, Mohammad Ghannaee Arani

**Affiliations:** 1 Trauma Research Center, Kashan University of Medical Sciences, Kashan, IR Iran

**Keywords:** Thorax, Complications, Wound and Injury, Mortality

## Abstract

**Background:**

Chest trauma is responsible for 50% of deaths due to trauma. This kind of death usually occurs immediately after the trauma has occurred.

**Objectives:**

The aim of this study was to evaluate the various aspects of chest trauma in patients admitted to two main trauma centers located in the Isfahan Province, Iran.

**Patients and Methods:**

During a six month period, a cross-sectional study of 100 patients who had sustained a chest injury was carried out. Data, including; age, sex, time of accident, trauma mechanism, organ injury, hospitalization length, complications, and patient outcomes were recorded and analyzed.

**Results:**

The mean ages of the patients were 24.7 ± 3.1 years. Men were injured four times more often than the women. Blunt trauma was the most common type of injury. The incidence of hemothorax was 38% in blunt traumas, and 45% (P = 0.01) in penetrating ones. The incidence of pneumothorax was 43% in blunt traumas and 20% in penetrating ones (P = 0.03). The length of hospitalization was 7.2 ± 3.2 days for blunt and 10.1 ± 3.1 days for penetrating chest traumas. Five patients (5%) died during hospitalization, three of those deaths were due to arterial injuries and two cases were due to lung contusions.

**Conclusions:**

Since hemothorax is the main complication of blunt chest trauma in young men, we recommend that special consideration needs to be made to any case of chest trauma, especially blunt chest injuries.

## 1. Background

Injury is one of the most frequent causes of death and complications in different societies and it is also considered to be the main cause of death in patients less than 40 years old (1). Chest trauma can be caused after blunt or penetrating traumas and can result in death in 25% of cases (2, 3). Most of these complications can be managed by using simple treatments such as a thoracostomy (4), however, in 10-15% of chest trauma cases, surgery is required (5). Death due to chest trauma composes half of all trauma deaths and usually occurs immediately after the chest injury (3). Increases in high speed road travel, and traffic violations have caused greater numbers of blunt chest trauma in the recent century (5). Blunt chest trauma is one of the most common problems experienced in road traffic accidents. Nevertheless, in spite of the benign nature of most trauma cases, there may also be an increased risk of mortality in such cases (4). Despite the fact that chest trauma is a significant issue, a complete survey on the current status of this condition, has not been performed in the central region of Iran.

## 2. Objectives

In order to access the current rate of chest trauma in Iran, more surveys need to be carried out in different parts of the country. For this purpose, a survey of 100 patients afflicted with chest trauma, who were admitted to two major trauma centers in the Isfahan Province, were investigated.

## 3. Patients and Methods

In a cross-sectional study, all of the chest trauma cases admitted to two major trauma centers in Isfahan Province, Iran, were studied over a six-month period. All chest trauma cases and rib fractures without complications generally undergo out-patient treatment, and these cases were excluded from this study. The necessary information regarding, age, sex, time of incident, cause and type of trauma according to the history according to the case notes, complications resulting from the trauma during hospitalization based on the history, physical examination, radiological information and findings during the operation, hospitalization period and consequences of the trauma, were gathered after the initial examinations and cardiopulmonary resuscitation (CPR) for each chest trauma case. The data was gathered by means of a check list and presented in the form of mean and standard deviations. Then, the data were analyzed with t-statistic tests. P-value, less than 0.05 was considered as meaningful.

## 4. Results

Out of 100 patients, 80 were male. The mean age of the patients was 24.7 ± 3.1 years. The most frequent age group affected was 20-30 (35%) year olds. The incidence in the other age groups is shown in Table 1. Sixty five percent of the trauma cases occurred between 12 midnight to 1 a.m. (65 patients). Sixty five percent of the patients suffered from blunt trauma. In the blunt trauma group, the most frequent cause of trauma was car accidents (72%), other cases included falling (5%), motorcycle accidents (3%) and fighting (2%). A total of 35 patients suffered from penetrating trauma, the most important factor in these cases was injury by stabbing which included 74% of the cases. Table 2 shows the complications of the trauma observed in the patients. According to the results of this table, hemothorax was the most frequent complication. Complications of chest trauma have been compared according to the type of trauma in Table 2. As can be seen in the results of this table, the incidence of hemothorax was 38% in blunt traumas and 45% (P = 0.01) in penetrating ones. The incidence of pneumothorax was 43% in blunt traumas and 20% in penetrating cases (P = 0.03). Tension pneumothorax was only observed in blunt trauma cases, and 34 of the patients suffered from rib fractures. In the majority of cases (72%), fractures occurred in the left ribs. Subcutaneous emphysema occurred in 51% and 9% of the penetrating and blunt traumas respectively (P > 0.05). Arterial injury, lung contusion and flail chest are other complications that accounted for, 0.7%, 0.3%, and 0.3% of the penetrating traumas respectively, and 0.6%, 1.3%, and 1.9% of blunt traumas respectively (P > 0.05). Hospitalization stay was 12 ± 3.2 days on average. The length of hospitalization was 7.2 ± 3.2 days for blunt and 10.1 ± 3.1 days for penetrating chest traumas. A total of 47% of the patients were hospitalized for less than 10 days, 12% for 10 days and 41% for more than 10 days. Head injuries affected 25% of the patients and 4% were accompanied by abdominal trauma. The most frequent complication of head trauma was linear fracture of the skull (72%) and the most frequent complication in abdominal trauma was liver trauma (60%). Five patients died during hospitalization, three of these deaths were due to arterial injuries and two cases were due to lung contusions, two cases suffered from arterial injuries which needed an emergency thoracotomy. In this study 4% of patients needed a laparotomy.

**Table 1:** Frequency Distribution of Injury by Age and Type

	Blunt, No. (%)	Penetrating, No. (%)	Frequency	*P *value
10 >, y	7 (11)	3 (8)	10	0.1
10-20, y	15 (24)	10 (28)	25	0.25
20-30, y	24 (37)	11 (36)	35	0.14
30-40, y	12 (19)	8 (23)	20	0.25
40-50, y	6 (7)	1 (4)	7	0.33
50 <, y	2 (2)	1 (1)	3	0.35

**Table 2:** Frequency Distribution of Injury by Complication

	Blunt, No. (%)	Penetrating, No. (%)	Frequency	*P* value
Hemothorax	25 (38)	16 (45)	41	0.01
Pneumothorax	28 (43)	7 (20)	35	0.03
Rib fracture	16 (10)	18 (51)	34	0.28
Subcutaneous emphysema	14 (9)	18 (51)	32	0.34
Arterial injury	1 (0.6)	2 (0.7)	3	0.5
Lung contusion	2 (1.3)	1 (0.3)	4	0.29
Flail chest	3 (1.9%)	1 (0.3)	4	0.4

## 5. Discussion 

In this present study, the sex distribution of chest trauma in the men was four times greater than for the women and consistent results have been found in similar studies (6, 7, 8, 9). Due to the fact that most chest trauma cases are the result of car accidents, the higher incidence of trauma in males may be due to a greater disregard of the driving laws by men. In addition, more men work outside and they may be exposed to more dangerous working conditions, which could be other reasons for the gender difference. The incidence rate of blunt trauma in this survey was 65%, but in a survey by Farooq et al. in Pakistan it was 8% (10), and in another survey by Basoglu et al. in Turkey it was 49% (11). The differences found in these studies may have arisen due to differences in the traffic culture in these societies. According to the results of this survey, one of the most frequent kinds of complications (after hemothorax and pneumothorax) in chest trauma cases are rib fractures. In a survey by Adegboye et al. rib fractures were also the most frequent complication which resulted from trauma (12). It seems that many rib fracture cases were examined as out-patients in Isfahan trauma centers or patients were treated without the need for hospitalization. Therefore, the findings are different from that of Adegboye. In blunt trauma cases it should also be taken into consideration that the probability of hemothorax will be greater than pneumothorax. Therefore, incidences of rib fractures on the left side are an issue that does not warrant further study. Subcutaneous emphysema is another complication of patients with chest trauma. Subcutaneous emphysema can have three different causes; spontaneous, traumatic or iatrogenic (13). The traumatic variety can occur secondary to rib fractures. Two cases of rib fractures in our study did not suffer from emphysema, however, 32 other patients were affected. Liman et al. (14) has found subcutaneous emphysema to be the most common finding on physical examination of trauma patients. In that study, 18% of the rib fractures ended in subcutaneous emphysema, but in our study 94% (32 out of 34) experienced subcutaneous emphysema. This may be due to lower vehicle safety standards and higher speed limits in Iran. It may also be the result of low seat belt usage and helmets for bikes. Insufficient pre-hospital care facilities before admission to the hospital may be another cause. The length of hospitalization for chest trauma cases was approximately 10 days, the same as reported by Basoglu et al. (11). The most frequent cause of trauma in this survey was car accidents (72%) and the most frequent complication was hemothorax, as has been mentioned by Haratian et al. (54.9%) (7), it appears that disregard of the driving laws is the main reason. Distribution of death frequency in this survey was 5%. Compared to other studies (12, 15), the rate in Iran is lower than that found in other countries and in order to find the main cause for this difference we should consider some other variables, such as the intensity of the trauma. In a study by Adegboye (12) this rate was 12% and in another study by Esme it was approximately 25% (15). In this present study, a laparotomy was required in 4% of the patients, a similar rate was also needed in the study by Esme et al.(15), however, more liver trauma was observed than spleen trauma in the present study. In the study by Esme (15), spleen trauma formed 62% of the abdominal trauma cases.

Finally, this survey shows that the majority of the chest trauma cases in Isfahan resulting from blunt trauma, are caused by road accidents. Therefore, obeying the traffic laws can reduce these kinds of trauma. Overall, this study shows that the most frequent kind of chest trauma was blunt ones which affect mainly males. The most frequent complication of trauma was hemothorax, while liver rupture was a frequent complication in the cases of abdominal trauma. The mortality rate of chest trauma found in this study was 5%. Because the rate is lower in Iran, more research need to be carried out in order to ascertain the reasons.

## References

[1] Nirula R, Pintar FA (2008). Identification of vehicle components associated with severe thoracic injury in motor vehicle crashes: a CIREN and NASS analysis.. Accid Anal Prev..

[2] Devitt JH, Pagliarello G, Simons J (1990). The involvement of anesthetists in critical care medicine.. Can J Anaesth..

[3] Vodicka J, Spidlen V, Safranek J, Simanek V, Altmann P (2007). [Severe injury to the chest wall--experience with surgical therapy].. Zentralbl Chir..

[4] Warner KJ, Copass MK, Bulger EM (2008). Paramedic use of needle thoracostomy in the prehospital environment.. Prehosp Emerg Care..

[5] Hines MH, Meredith JW, Ritchie WP, Steele G, Dean RH (1995). Special problems of thoracic trauma.. General surgery..

[6] Falah P, Shafigh Y, Jeddi SN, Yadegari S (2004). Management of asymptomatic patients following stab wound to the chest.. J Qazvin Uni Med Sci..

[7] Haratian Z, Zareei S, Lashkari M (2005). Surveying the frequency of chest trauma (blunt and penetrating in Air Force Hospital, 2002-2004.. Med Sci J Islamic Azad Uni..

[8] Inan M, Ayvaz S, Sut N, Aksu B, Basaran UN, Ceylan T (2007). Blunt chest trauma in childhood.. ANZ J Surg..

[9] Zargar M, Khaji A, Karbakhsh Davari M (2007). Thoracic injury: a review of 276 cases.. Chin J Traumatol..

[10] Farooq U, Raza W, Zia N, Hanif M, Khan MM (2006). Classification and management of chest trauma.. J Coll Physicians Surg Pak..

[11] Basoglu A, Akdag AO, Celik B, Demircan S (2004). [Thoracic trauma: an analysis of 521 patients].. Ulus Travma Acil Cerrahi Derg..

[12] Adegboye VO, Ladipo JK, Brimmo IA, Adebo AO (2002). Blunt chest trauma.. Afr J Med Med Sci..

[13] Salib RJ, Valentine P, Akhtar S (1999). Surgical emphysema following dental treatment.. J Laryngol Otol..

[14] Oliver AJ, Diaz EM, Jr., Helfrick JF (1993). Air emphysema secondary to mandibular fracture: case report.. J Oral Maxillofac Surg..

[15] Esme H, Solak O, Yurumez Y, Yavuz Y, Terzi Y, Sezer M (2007). The prognostic importance of trauma scoring systems for blunt thoracic trauma.. Thorac Cardiovasc Surg..

